# Therapies for Heart Failure: Clinical Updates and Perspectives

**DOI:** 10.3390/jcm15176624

**Published:** 2026-08-27

**Authors:** Stela Iurciuc, Dragoș Cozma, Cristina Tudoran

**Affiliations:** 1 Research Center of the Institute of Cardiovascular Diseases, 13A Gheorghe Adam Street, 300310 Timisoara, Romania; iurciuc.stela@umft.ro; 2 Department of Internal Medicine and Cardiovascular Prevention, “Victor Babeș” University of Medicine and Pharmacy, E. Murgu Square, Nr. 2, 300041 Timisoara, Romania; 3 Department of Cardiology, Railway Clinical Hospital, 300643 Timisoara, Romania; 4 Cardiology Clinic, Institute of Cardiovascular Diseases, 300310 Timisoara, Romania; 5 Department of Cardiology, “Victor Babeș” University of Medicine and Pharmacy, E. Murgu Square, Nr. 2, 300041 Timisoara, Romania; 6 Department VII, Internal Medicine II, Discipline of Cardiology, “Victor Babeș” University of Medicine and Pharmacy, E. Murgu Square, Nr. 2, 300041 Timisoara, Romania; 7 Department of Cardiology, County Emergency Hospital “Pius Brînzeu”, 300723 Timisoara, Romania; 8 Centre of Molecular Research in Nephrology and Vascular Disease, “Victor Babeș” University of Medicine and Pharmacy, E. Murgu Square, Nr. 2, 300041 Timisoara, Romania

Although signs and symptoms of heart failure (HF) have been described since ancient times, and the term has evolved throughout history, there are still unanswered questions concerning its pathophysiology and best treatment options. The European Society of Cardiology provides dedicated, regularly updated guidelines for the management and treatment of HF [1,2,3,4,5]. The treatment of HF is very complex and not limited to drug therapy [6]. This reprint presents groundbreaking research into the causation and treatment of HF. This reprint is divided into four main parts.

The first part of “Therapies for Heart Failure: Clinical Updates and Perspectives” describes the mechanisms underlying HF, which could serve as targets for new therapeutic options [7,8,9]. Inflammation is a major pathophysiological mechanism responsible for the occurrence of acute coronary syndrome (ACS), contributing to atherosclerotic plaque destabilization [10], myocardial injury [11], and adverse clinical outcomes [12,13,14,15,16]. Inflammatory biomarkers, together with N-terminal pro–B-type natriuretic peptide (NT-proBNP), are increasingly used for risk stratification, yet their prognostic value in different ACS phenotypes remains unclear [17,18,19]. An unfavorable clinical profile, characterized by older age, impaired renal function, and reduced left ventricular systolic function, was associated with major adverse cardiovascular events (MACE) during index hospitalization in patients with ACS. Levels of inflammatory biomarkers (high-sensitivity C-reactive protein (hs-CRP), neutrophil-to-lymphocyte ratio (NLR), and systemic immune-inflammation index (SII)) and NT-proBNP were significantly higher among those who developed in-hospital MACE compared with those without events, with CRP showing the strongest association. These findings suggest that inflammatory biomarkers characterize the inflammatory status present in ACS and may provide complementary information regarding the inflammatory burden, whereas their incremental prognostic contribution beyond established clinical variables remains to be further clarified [17].

The research by Pah et al. suggests that systemic inflammation, characterized by elevated interleukin 6 (IL-6), hs-CRP, and NLR, is a strong and independent predictor of mortality and adverse outcomes in HF [20]. These biomarkers capture distinct yet complementary dimensions of the inflammatory cascade and provide additive prognostic value beyond established clinical and neurohormonal indicators. IL-6 stands out as the most consistent and biologically plausible marker, supporting its dual role as both a mechanistic driver and a potential therapeutic target [21,22,23].

The second part focuses on associated comorbidities. Frailty is a major determinant of adverse outcomes in older adults with HF, and is often difficult to assess, especially in patients with acute HF [24,25,26]. Garcia et al. compared the manual short Physical Performance Battery (mSPPB) with an automated electronic SPPB (eSPPB) system to evaluate frailty in patients with decompensated cardiovascular disease (CVD). The automated eSPPB demonstrated good agreement with manual assessment and was significantly associated with 30-day mortality in hospitalized patients with HF, but these findings should be interpreted with caution and confirmed in larger multicenter studies before routine clinical use [27].

The COVID-19 pandemic posed new and unexpected challenges for cardiologists who managed patients with CVD and HF. Several studies demonstrated that CVD and systemic hypertension (SH) are associated with a worse prognosis in patients with COVID-19 [28,29]. In a single-center study, the combined presence of SH and pre-existing HF identified patients at increased risk for in-hospital mortality, admission to the intensive care unit, and prolonged hospitalization. This study concluded that the risk for developing complications during a COVID-19 infection was elevated in patients suffering from both AH and HF compared to those without cardiovascular comorbidities or with only SH [30]. A combined cardiovascular burden might be associated with increased inflammatory activation, as evidenced by elevated interleukin-6 levels at hospital admission. These findings highlight the clinical significance of systemic inflammation as a driver of disease severity in patients with preexisting cardiovascular conditions [30].

During this pandemic, physicians discovered that patients with HF infected with the Severe Acute Respiratory Syndrome Coronavirus 2 (SARS-CoV-2) had a higher incidence of unfavorable outcomes [31,32,33]. In hospitalized COVID-19 patients, pre-existing HF identifies a subgroup with heightened cardiac biomarker activation and a substantially higher burden of cardiovascular complications, but does not associate with sepsis or short-term mortality in this cohort after adjustment for inflammatory and cardiac biomarkers [30]. Inflammatory markers, particularly CRP and interleukin-6, together with high-sensitivity troponin, were independently associated with mortality in adjusted analyses, highlighting the central role of dysregulated host immune response and myocardial injury as proximal drivers of adverse outcomes. By contrast, NT-pro BNP and a prior diagnosis of HF reflected baseline myocardial stress and structural vulnerability, yet did not provide incremental prognostic information once dynamic biomarker profiles were considered [30].

The third part of “Therapies for Heart Failure: Clinical Updates and Perspectives” describes management and treatment options for HF. Acute decompensated heart failure (ADHF) is a cardiac emergency and often raises challenges for the treating physician, especially due to its complexity and associated comorbidities [34,35,36,37,38]. In a large, national cohort of adults hospitalized with ADHF, collected from the 2016–2020 Nationwide Inpatient Sample (NIS), weekend admission was associated with higher in-hospital mortality and increased odds of major complications, including cardiac arrest, compared with weekday admission. These results are consistent with the broader literature documenting a weekend effect across multiple conditions and settings, and with contemporary HF data highlighting vulnerability during off-hours [39,40,41,42].

HF represents a complex and heterogeneous syndrome that extends beyond myocardial dysfunction, encompassing dynamic interactions between cardiac, vascular, and systemic factors [43,44,45]. The integration of vascular function into precision medicine frameworks offers an opportunity to refine phenotypic classification and the understanding of the pathophysiological mechanisms of this disease. Arterial stiffness, as assessed by pulse wave velocity, represents a robust and physiologically relevant marker of vascular aging and cardiovascular risk. Its incorporation into multimodal phenotyping strategies may enhance risk stratification and support the identification of clinically meaningful subgroups within the heterogeneous spectrum of HF [46,47,48,49].

Contemporary guidelines [1,2] recommend rapid initiation of the four “pillars” of guideline-directed medical therapy (GDMT) for HF with reduced ejection fraction (HFrEF). In recent years, Sodium-Glucose Cotransporter 2 inhibitors (SGLT2i) have established themselves as one of the four “pillars” for the treatment of HF [1,2,50]. A multidomain synthesis demonstrates that SGLT2 inhibitors provide consistent, robust reductions in mortality and hospitalizations, while also improving patient-reported health and functional status. Improvements in symptoms, quality of life, and cardiac remodeling are evident, but these results should be interpreted with caution, especially for acute HF and HF with preserved ejection fraction (HFpEF) [51].

In a study conducted on 1363 outpatients with HFrEF, with at least two years of follow-up, Lopez et al. demonstrated that the systematic introduction of ARNI and SGLT2i in the treatment of HFrEF was associated with improved ventricular function, reduced need for device implantation, and a significant improvement in two-year survival [52]. This benefit was independent of the baseline clinical characteristics of these patients. These data suggest that the systematic application of therapies guided by evolving guidelines and active titration strategies resulted in superior clinical outcomes and improved ventricular recovery [52].

By analyzing 27 studies and 73,174 patients with HF, Gómez-Ochoa et al. observed that a more complete GDMT at hospital discharge was associated with a lower cardiovascular event rate, with quadruple therapy having the best results on HF outcomes. Unfortunately, their data showed a suboptimal prescription of all four pillars of DDMT at discharge of patients hospitalized for HF [53]. On the other hand, real-world persistence, adherence, and dose optimization remain suboptimal. Data from a non-randomized sub-registry within the prospective Cardiology Research Dubrava (CaRD) registry evidenced that although in-hospital initiation of quadruple GDMT in patients with newly diagnosed HFrEF was feasible, maintaining long-term adherence and achieving target doses remained challenging. These data underscore the gap between guideline recommendations and routine practice and support structured follow-up and protocol-driven titration to optimize implementation [54].

The fourth part of this reprint suggests future perspectives for the treatment of HF.

Future prospective multicenter studies, stratification by HF phenotype, incorporation of inflammatory and cardiac biomarkers, arterial stiffness parameters, and associated comorbidities are needed to improve the assessment of HF [30,46,55]. Future research integrating myocardial, vascular, and systemic domains through advanced computational approaches is likely to redefine HF classification and pave the way toward truly personalized cardiovascular medicine [46]. Such approaches may enable earlier identification of high-risk patients and facilitate the development of customized therapeutic strategies that target both immune dysregulation and myocardial injury in severe COVID-19 [30,56,57]. Interventional research should explore whether targeted cardioprotective strategies, optimized guideline-directed HF therapy, and anti-inflammatory approaches (including IL-6–directed or broader immunomodulatory regimens) can mitigate the excess risk observed in patients with COVID-19 and concomitant AH and HF [30,58].

Structured follow-up, repeated patient education, and protocolized titration pathways may be required to sustain quadruple therapy and optimize dosing over time in HRrEF [54,59,60,61,62,63,64].

Further dedicated mechanistic studies in HFpEF and acute HF are needed to refine phenotype-specific understanding and to clarify the long-term structural and functional implications of treatment with SGLT2i [51,65,66,67]. Applying standardized HF pathways, strengthening weekend coverage, and expanding 7-day diagnostic and critical-care services may help mitigate the weekend effect and promote more unbiased outcomes for patients hospitalized with ADHF, regardless of the day of admission [39,68,69].

Adequately powered randomized trials of protocolized in-hospital quadruple initiation are therefore the key next step to establish the incremental benefit of this approach and to close the gap between evidence and practice [53,70].

## Data Availability

Data are contained within the article.
